# Two-Stage Deep-Learning Classifier for Diagnostics of Lung Cancer Using Metabolites

**DOI:** 10.3390/metabo13101055

**Published:** 2023-10-07

**Authors:** Ashvin Choudhary, Jianpeng Yu, Valentina L. Kouznetsova, Santosh Kesari, Igor F. Tsigelny

**Affiliations:** 1 School of Life Science, University of California, Los Angeles, CA 90095, USA; ashvin101@g.ucla.edu; 2 School of Computer Science, Carnegie Mellon University, Pittsburgh, PA 15213, USA; jianpeny@alumni.cmu.edu; 3 San Diego Supercomputer Center, University of California, San Diego, CA 92093, USA; vkouznetsova@ucsd.edu; 4 IUL, La Jolla, CA 92038, USA; 5 CureScience Institute, San Diego, CA 92121, USA; 6 Pacific Neuroscience Institute, Santa Monica, CA 90404, USA; santosh.kesari@providence.org; 7 Department of Neurosciences, University of California, San Diego, CA 92093, USA

**Keywords:** lung cancer, machine learning, metabolites

## Abstract

We developed a machine-learning system for the selective diagnostics of adenocarcinoma (AD), squamous cell carcinoma (SQ), and small-cell carcinoma lung (SC) cancers based on their metabolomic profiles. The system is organized as two-stage binary classifiers. The best accuracy for classification is 92%. We used the biomarkers sets that contain mostly metabolites related to cancer development. Compared to traditional methods, which exclude hierarchical classification, our method splits a challenging multiclass task into smaller tasks. This allows a two-stage classifier, which is more accurate in the scenario of lung cancer classification. Compared to traditional methods, such a “divide and conquer strategy” gives much more accurate and explainable results. Such methods, including our algorithm, allow for the systematic tracking of each computational step.

## 1. Introduction

The field of machine learning (ML) and deep learning is growing in many disciplines. ML is widely applied to medicine and pharmacology. In this study, we implemented ML in the early diagnosis of lung cancer by identifying distinctions in the metabolic profiles of each type of lung cancer.

Lung cancer is the leading cause of cancer deaths in the U.S. with an estimated 127,070 deaths in 2023. One in sixteen people will be diagnosed with lung cancer in their lifetime. In fact, the prognosis of lung cancer is poor, with a five-year survival rate after the diagnosis (2012–2018) of only 22.9% [1]. Such poor prognosis is correlated with the fact that there is a lack of early detection methods and the difficulty of treating metastatic lung cancer.

**Figure 1:**
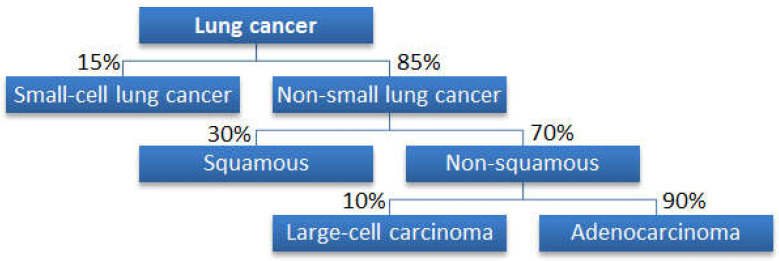
Classification of lung cancer by histopathologic subtype (reproduced from open source [2]).

The taxonomy of lung cancer by histopathologic subtype is shown in Figure 1 [2]. There are two major categories: non-small-cell lung cancer (NS) and small-cell lung cancer (SC) [2,3]. Within the umbrella of NS, there are three subtypes: adenocarcinoma (AD), squamous cell carcinoma (SQ), and large-cell lung cancer (LC) [2,3]. Anatomically, AD usually begins to occur on the outer part of the lungs and is the most common form of NS [3]. AD is histopathologically subdivided into several different subtypes, which we omitted from our analysis due to the lack of data. SQ usually occurs near the chest in the bronchi [3]. Finally, LC is the least common type of lung cancer and is the cancer with the highest chance of becoming malignant [3]. The other major category of lung cancer, SC, is very aggressive, and the cancer grows rapidly, unlike in the other subtypes [4].

Since lung cancer can come in so many types, it makes it hard for doctors and radiologists to effectively diagnose it at early stages and beyond. For example, it is crucial for medical staff to be able to differentiate between AD and SQ because the chemotherapy regimens for each cancer are different [5]. Thus, being able to identify the type of lung cancer at an early stage could aid in the early planning of treatments specific to the type of lung cancer.

Traditional methods of screening and detecting lung cancer are chest X-rays and CT-scans. However, both methods heavily depend on the radiologist’s opinion, which could foster observer s [6,7]. Metabolites, on the other hand, can act as biomarkers and can provide safe and noninvasive method. Using ML, the metabolic profiles could act as datasets to help in the early recognition of lung cancer to distinguish different types of vs control group. For example, Mazzone and colleagues have shown differences in the metabolite profile between patients of NS and healthy individuals [8]. The study concluded effective results displaying C statistics between 0.75 and 0.85 [8], however, authors did not run an independent test set. In other attempts by Kouznetsova and colleagues, the studies utilized ML models with metabolite profiles to classify the stages of bladder cancer with 82.54% accuracy [9] and deep learning to distinguish oral cancer from periodontal diseases with accuracy 79.57% [10]. Wu and colleagues developed a ML model using metabolite biomarkers for diagnostics of lung cancer [11]. In the study, they could differentiate the lung cancer patients from healthy individuals or even patients with tuberculosis, which has similar symptoms, at an accuracy of 95.7%. Fahrmann and colleagues, in their study, correlated eight specific metabolite biomarkers as candidates for diagnosing AD versus a control [12]. After creating a machine-learning model on the eight metabolites, the study ran an independent test set which resulted in a 77.3% accuracy. In our project we developed a strategy to distinguish four types of lung cancer.

## 2. Methods

**Figure 2:**
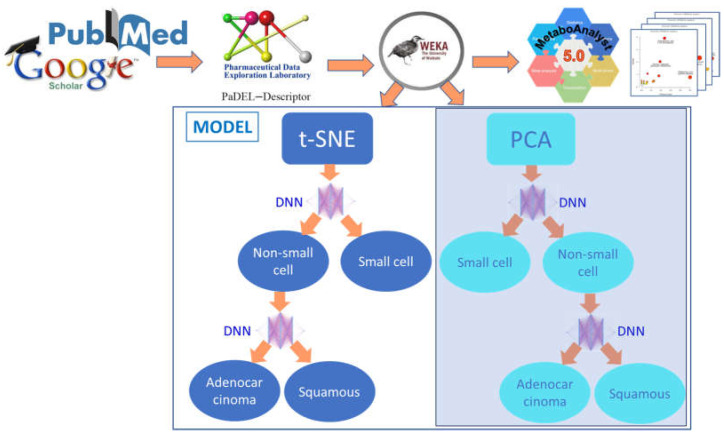
Overview of the methods. The PCA and t-SNE were applied to categorize NS and SC. The experiments show that t-SNE has a better result with 92.0% overall accuracy. The t-SNE gives more accurate results to distinguish NS/SC (96.1% vs. 92.3%) and AD/SQ (91.1% vs. 84.6%) compared to the PCA Model. More explanations can be found in the Section 3.

We used metabolites from the four types of lung cancer (SQ, NS, SC, and AD) and created a classification algorithm to distinguish between the four types of lung cancer. The algorithms were designed based upon the current lung cancer taxonomy (Figure 1) [2]. Figure 2 illustrates the flowchart of the algorithm’s steps. Because AD and SQ are subtypes of NS, first, we used a neural network to distinguish between the NS and SC cancer. If the metabolite fits the NS cancer profile, then we used another neural network to distinguish between AD and SQ.

The general flowchart of methods is shown in Figure 2, and it also displays a classification algorithm. To select the best dimensionality reduction method, we tested PCA and t-SNA methods and found that t-SNA gave better accuracy in some tasks. So, we used these methos correspondingly where they have better results (see Section 3). In short, our model first applies t-SNE dimensionality reduction, then creates two classification tasks, namely NS/SC and AD/SQ, each of which is performed by a DNN network. Combining two tasks will result in the final cancer type. The model was implemented using Python.

The model converted SMILE structures for each metabolite to molecular descriptors. Then, the important descriptors were selected by InfoGainAttributeEval (InfoGain). For training purposes, the training datasets were used for each lung cancer type. For testing the created classification system, we used the completely independent set of metabolites related to the studied cancers. After running 5-fold cross-validation several times, we found that accuracy drops to about 40% for selecting metabolites with an FC of less than 1 or above 1.5. We eventually chose 1.2 as the threshold for the fold change (FC) for all types of lung cancers including NS, SC, AD, and SQ. All the datasets were filtered by *p* ≤ 0.05 and FC ≥ 1.2.

To make sure that the selected metabolites are not random but are the biomarkers of the specific cancer’s development, we conducted the elucidation of the metabolic pathways with the pathway enrichment analysis module of MetaboAnalyst software [13].

### 2.1. Datasets for Cancer Classifications

All data were retrieved from public sources and filtered by *p* values lower than 0.05 and a fold change (FC) greater than 1.2 (*p* < 0.05, FC > 1.2).

The non-small-cell lung cancer (NS) training data were obtained from Mazzone and colleagues’ non-small-cell lung cancer set collected from the sera of cancer patients [8]. After filtering the dataset, 38 total distinct metabolites were left. The independent test set for NS was retrieved from the serum metabolites set collected by Chen and colleagues [14].

The small-cell lung cancer (SC) data were retrieved using Wedge and colleagues’ dataset of metabolites from cancer patients’ sera [15]. After filtering the dataset, 35 total distinct metabolites were left. The independent test set for SC was collected from Li Yu and colleagues [16].

The adenocarcinoma (AD) data were extracted from Mazzone and colleagues’ dataset of metabolites from the sera of cancer patients [8]. After filtering the dataset, 44 total distinct metabolites were left. The independent test set for AD was obtained from the publication of Fahrmann and colleagues [12].

The squamous cell carcinoma (SQ) data were acquired from Mazzone and colleagues’ dataset of metabolites from the sera of cancer patients [8]. After filtering the dataset, 46 total distinct metabolites were left. The independent test set for SQ was collected from Liu and colleagues’ metabolite set from the sera of cancer patients [17]. The metabolites used in the training are presented in Table S1.

### 2.2. MetaboAnalyst

MetaboAnalyst is an online software for metabolomics data analysis. With MetaboAnalyst, a user can complete statistical analysis, functional analysis, meta-analysis as well as integrative analysis with other omics data [13]. It has modules for different statistical analyses, biomarker analyses, enrichment and pathway analyses, and joint gene–metabolite pathway analysis. Pathway enrichment analysis allows the identification of metabolites with similar functions and the interpretation of their patterns in light of metabolomic pathways. We elucidated the metabolic pathways that produce the metabolites used in our calculation. Our analysis demonstrated that these metabolites are not random but are related to cancer development.

### 2.3. Raw Data and Physiochemical Descriptors

The general flowchart of our study is presented in Figure 2. The data were collected from the aforementioned public sources and were filtered by *p* values and fold change. Then, SMILES nominations of compounds were elucidated and used to assign them to PaDEL descriptors [18]. After the PaDEL descriptors were calculated, the data subtypes were split into two different classifications. The first classification was between NS and SC (classification 1). The second classification was between AD and SQ (classification 2). A sufficient dataset for rare LC was not found, so it was not included in the classification system. All PaDEL descriptors for each classification were then normalized in the range between 0 and 1, using the formula below [19] (Equation (1)):

(1) $$x=\frac{x-\min\left(x\right)}{\max\left(x\right)-\min\left(x\right)},$$

where *x* = numerical value of the descriptor.

### 2.4. Discretization and InfoGain

After data normalization, we ran InfoGain, a WEKA filter, to see which features contain the most information [20]. In information gain, the first step is to discretize the data into different bins. Each bin represents one ordinal category in InfoGain. Then, we calculate the information gain for each pair of features “*a*” and category “*I*”. Then, we sum up all the ordinal categories via the second equation below [21]:

(2) $$IG\left(T,a\right)=H\left(T\right)-H\left(T|a\right),$$

where

(3) $$H\left(T\right)=\sum_{i=0}^{n}-P\log\left(Pi\right),$$

where *IG*(*T*, *a*) is the information gain by choosing attribute *a*; *H*(*T*) is the cross-entropy of the label without choosing feature a; and *H*(*T*|*a*) is the cross-entropy of the label after choosing the features. Before information gain, there were 1083 PaDEL features for each metabolite. But, after InfoGain filtration, only 170 features were selected for each metabolite, which lowered the noise in the data.

### 2.5. Dimensionality Reduction

To transform the data from a high-dimensional space into a low-dimensional space—dimensionality reduction—two algorithms were used: (1) principal component analysis (PCA) [22] and (2) t-distributed stochastic neighbor embedding (t-SNE) [23,24]. PCA is one of the most common linear methods to reduce data dimensionality. To perform PCA, a co-variance matrix (C*ov*(*X*–*E*(*X*))) should first be calculated for *m*-dimensional data *X* (where *E*(*X*) is the average expectation of data *X*), defined as [22,23]:

(4) $$Cov\left(X\right)=\left(X-E\left(X\right)\right){\left(X-E\left(X\right)\right)}^{T}.$$

After computing the co-variance matrix, m dimensional data could be reduced to *n*, *n* ≤ *m*, by selecting the first n eigenvectors of *Cov*(*X*) as output. All steps used in PCA are linear transformations, and therefore, PCA works best with linear input data. In contrast, t-SNE is a stochastic method used for nonlinear data, reducing the high-dimensional set *X* to a two- or three-dimensional set *Y* of vectors ***y****_i_*, using conditional probabilities as similarities [23,24]. Let us have a set of *N* high-dimensional data *x*_1_, *x*_2_, *x*_3_, … *x_N_*; *x_i_* ∈ ℝ*^k^* that have to be transformed into a set of low-dimensional data *y*_1_, *y*_2_, *y*_3_, … *y_N_*; yi ∈ ℝ^d^, where dimension *d* = 2 or 3. With t-SNE, a Gaussian distribution of similarity probabilities is first computed. The similarity probability Pr between two objects, namely *x_i_* and *x_j_* (*I* ≠ *j*), is defined as [23]:

(5) $$\mathrm{Pr}\left(i\left|j\right|,i\neq j\right)=\frac{\exp\left(-\frac{{\left|\left|x_{i}-x_{j}\right|\right|}^{2}}{2\sigma_{i}^{2}}\right)}{\sum_{k\neq i}\exp\left(-\frac{{\left|\left|x_{i}-x_{k}\right|\right|}^{2}}{2\sigma_{i}^{2}}\right)}$$

where *σ_i_* is the variance of the Gaussian centered around *x_i_*

(6) Pr⁡(i | i)=0

(7) $$\mathrm{and}\;\sum_{j}Pr(i\;|\;j)=1,\;\forall i\;.$$

Note that Pr(*x*|*y*) might not be equal to Pr(*y|x*). Therefore, t-SNE defines a mutual similarity score *S_ij_* such that *s_ij_* = *s_ji_:*

(8) $$s_{ij}=\frac{Pr\left(\mathrm{i}|j\right)+Pr\left(j|\mathrm{i}\right)}{2N}.$$

In other words, *s_ij_* can be considered an average of both Pr(*j*|*i*). and Pr(*i*|*j*) normalized by 1/*N*.

To reduce the dimension, Student’s t-distribution is used, which allows us to fit the information of high-dimensional data in the low-dimensional embedding space (usually 2 or 3). t-SNE tries to learn a lower d dimensional distribution y, y ∈ R^d^ that preserves similarity scores *s_ij_* as much as possible. A similarity score for y might be defined as *Q*, *q_i_* = *q_ji_*: [23]

(9) $$Q_{ij}=\frac{{\left(1+{\left\|y_{i}-y_{j}\right\|}^{2}\right)}^{-1}}{\sum_{k}\sum_{l\;\neq k}{\left(1+{\left\|y_{l}-y_{k}\right\|}^{2}\right)}^{-1}}.$$

t-SNE uses the metric of the Kullback–Leibler divergence (KL-divergence) to compare two distributions. In a short sentence, one finds the d-dimensional distribution *Q* minimizing the KL-divergence between itself and the original k-dimensional distribution *S* [25]:

(10) $$argmin_{Q}KL(S||Q)=argmin_{Q}\sum_{i!=j}s_{ij}\log\left(\frac{s_{ij}}{Q_{ij}}\right).$$

### 2.6. Dimensionality Reduction for NS/SC

Because, in this project, we split the classification into two subsets, we applied two different dimensionality reduction methods to them. After visualizing the t-SNE and PCA dimensionality reduction method, the results of which are shown in graphs, we observed that data for NS/SC are much more nonlinear than AD/SQ (see the Results section). Thus, for NS/SC classification, we applied the t-SNE dimensionality reduction method. Results are presented below in the Results section. t-SNE will first compute a probability matrix *p_ij_* for the original data and then another probability matrix *q_ij_* in the lower dimension, minimizing the KL divergence between two distributions. In contrast to the linear PCA method, t-SNE calculates a nonlinear lower dimensionality representation of the original high-dimensional space. Therefore, we ran an t-SNE dimensionality reduction method for NS/SC classification.

### 2.7. Dimensionality Reduction for AD/SQ

After visualizing the t-SNE and PCA dimensionality reduction method results on the graphs shown in the Results section, we observed that both NS/SL and AD/SQ were nonlinear, so we applied t-distributed stochastic neighbor embedding (t-SNE). Compared to the PCA method, t-SNE subjects the data to nonlinear transformation and selects features from eigenvectors of the linear co-variance matrix. In effect, for AD/SQ, we used the t-SNE reduction method.

### 2.8. Design for the Neural Networks

**Figure 3:**
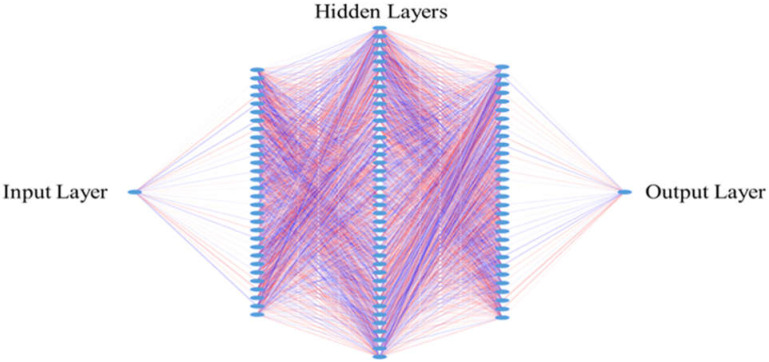
Neural network used in both NS/SC and AD/SQ classification.

In the second step, for each classification (NS/SC and AD/SQ), we applied a three-layer deep neural network (DNN) classifier shown in Figure 3, and each layer has 300, 400, and 300 neurons, respectively. The numbers of neurons are optimized using the grid search technique. Grid searches explore all possible combinations of numbers of neurons in each layer and return the combination with best accuracy performance. The neural net was connected in the simple feedforward fashion and there are no backward edges unlike long short-term memory (LSTM) networks (Figure 3). We used Adam optimizer to train our neural networks for faster convergence instead of the stochastic gradient descent method [26].

### 2.9. Hyperparameters

In this project, we performed a grid search to select the best hyperparameters. The following table gives the selected hyperparameters. We included a 0.5 dropout rate to overcome overfitting. A dropout of 0.5 means that each neuron and its connections will have a 50% rate to be randomly excluded in the calculation. As a result, the overall neuron network will be a statistical average of a set of sampled nets. Instead of the stochastic gradient descent (SGD) approach, herein, we used the Adam optimizer, the extended version of SGD [26]. Adam computes the bias-corrected two-moment estimates and accordingly updates the parameters of the neural network. Compared to the SGD method, Adam gives a much faster convergence. We used a relatively medium learning rate (0.01) and let it exponentially decay. After each step s, the learning rate will multiply by the decay rate 0.96 before it reaches a maximum of decay steps of 10,000. The formula for the learning rate *lr* is

(11) $$lr=\left\{\begin{array}{c} 0.01\times 0.96^{s}\;0\leq s\leq 10000\\ 0.01\times 0.96^{10000}\;s\geq 10000 \end{array}\right.$$

Exponential decay allows our neural network to converge faster because initially a relatively large learning rate is desired to accelerate the training and, in the end, a smaller learning rate is desired; so that a stable result will be obtained|. The rest of our hyperparameters are listed in Table 1.

## 3. Results

**Figure 4:**
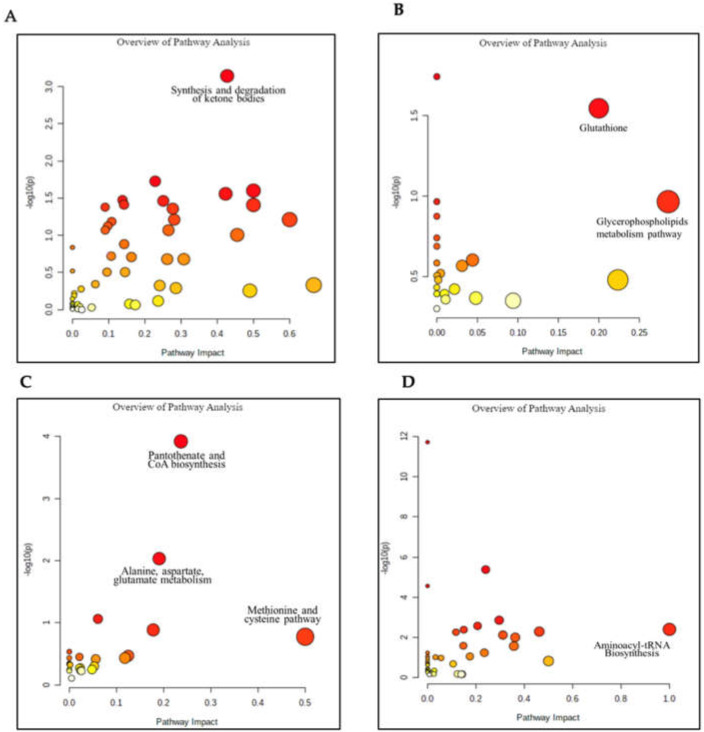
Pathways were elucidated by MetaboAnalyst and presented in a diagram. The position on the Y axis and vibrancy in color are based on the *p*-value, while the position on the X axis and the size of the point represent the pathway’s impact values. The significance of the pathway plots for each cancer are given: (**A**) is for non-small-cell lung cancer; (**B**) is for adenocarcinoma; (**C**) is for squamous cell carcinoma; (**D**) is for small-cell carcinoma.

Using MetaboAnalyst, we found the most significant pathways for each type of lung cancer, which are illustrated in plots of Figure 4.

### 3.1. Important Pathways for Lung Cancers

#### 3.1.1. Important Pathways for Non-Small-Cell Lung Cancers 

The synthesis and degradation of the ketone bodies pathway provides an energy source for the cell [27] (Figure 4A). When fatty acids are broken down, these produce a water-soluble byproduct called ketone bodies. Tumor cell proliferation and immune system response require massive energy, and therefore, a ketone body will be generated from the β oxidation of fatty acids to provide energy for cancer cells. Recent positron emission tomography research shows that an anticancer immune response from macrophage cells will consume more glucose and trigger beta-oxidation when glucose is insufficient [28].

#### 3.1.2. Important Pathways for Adenocarcinoma Lung Cancers 

The glycerophospholipid metabolism pathway (Figure 4B) helps create the cellular membranes, which hold the organelles of the cell [29]. The glycerophospholipids are valuable in creating the lipid bilayer in all cells. This pathway is vital as cancer cells need to increase the synthesis of glycerophospholipids to meet the standards for membrane production [30].

Glutathione (GSH) is the most abundant antioxidant used to detoxify the cells of carcinogens and radicals [31]. Excessive GSH promotes tumor progression and metastasis because GSH will protect tumor cells from oxidation in rapid tumor cell proliferation [32]. 

#### 3.1.3. Important Pathways for Squamous Cell Lung Cancers 

The pantothenate and CoA biosynthesis pathway (Figure 4C) is valuable for a variety of reasons in the cell. Pantothenate, or vitamin B, is the precursor for the synthesis of CoA [33]. CoA itself is valuable for cell growth as it is involved in many metabolic pathways like the synthesis of phospholipids and the synthesis/degradation of fatty acids [33]. Since this pathway deals with the synthesis/degradation of fatty acids, the pantothenate and CoA biosynthesis pathway could be vital for cancer cells to generate the necessary energy to survive.

The methionine and cysteine pathway (Figure 4C) comprises sulfur-containing amino acids that are critical to the production of significant protein structures and metabolism in the cell [34]. Cancer cell proliferation requires proteins containing disulfide bonds and methionine is an essential precursor for disulfide bonds.

Alanine, aspartate, and glutamate metabolism (Figure 4C) constitute an important pathway producing the three amino acids. Alanine is an important precursor for the breakdown of tryptophan and vitamin B6. Alanine is also broken down for energy in muscle and in the central nervous system. Glutamate is a neurotransmitter that helps send signals from one nerve cell to the next nerve cell. Aspartate is a valuable metabolite for preserving the membrane potential in the mitochondria needed to produce energy for the cell [35]. All three metabolisms are required by cancer cells to provide enough energy for tumor cell growth.

#### 3.1.4. Small-Cell Carcinoma Pathways

Aminoacyl-tRNA biosynthesis (Figure 4D) is an important pathway that creates aminoacyl-tRNA, which helps convert the genetic code of mRNA into an amino-acid chain for the production of protein [36]. The aminoacyl-tRNA biosynthesis pathway helps cancer cells create proteins necessary for the survival of that cell [36].

### 3.2. Dimensionality Reduction

**Figure 5:**
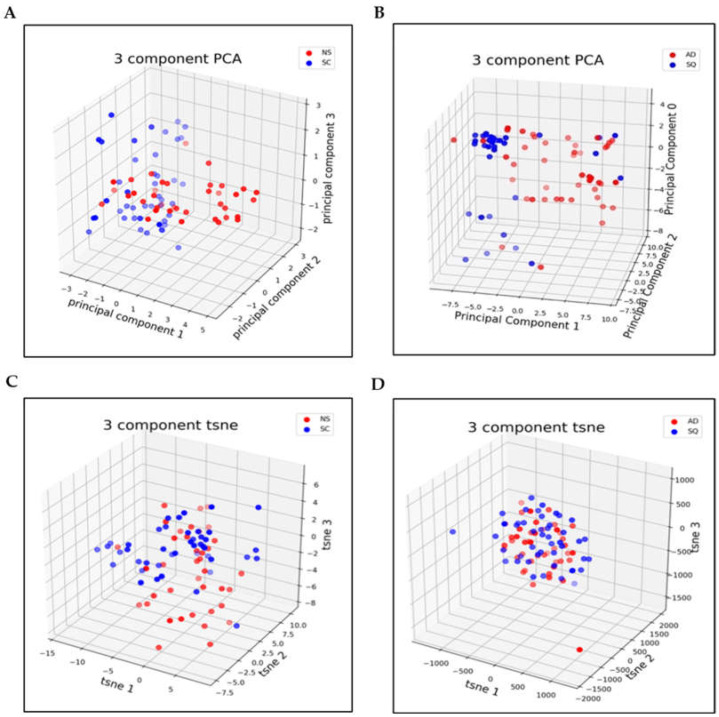
Three-dimensional visualization of the PCA and t-SNE components after dimensionality reduction (**A**): 3-component PCA method for NS/SC classification; (**B**): 3-component PCA method for AD/SQ classification; (**C**): 3-component t-SNE method for NS/SC classification; and (**D**): 3-component t-SNE method for AD/SQ classification.

**Figure 6:**
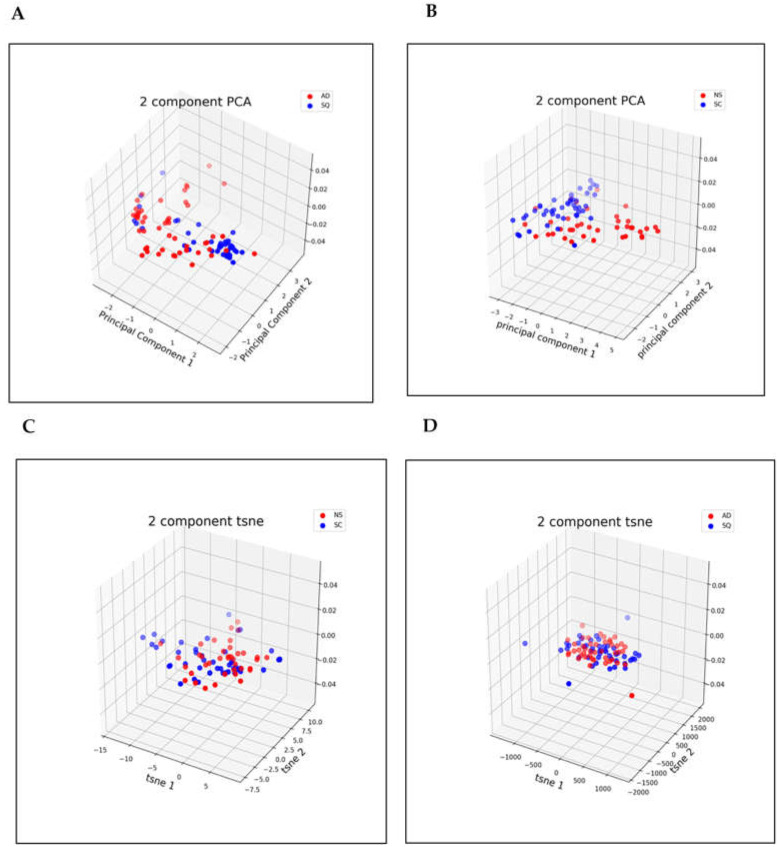
Two-dimensional visualization of PCA and t-SNE components after dimensionality reduction (**A**): 2-component PCA method for NS/SC classification; (**B**): 2-component PCA method for AD/SQ classification; (**C**): 2-component t-SNE method for NS/SC classification; and (**D**): 2-component t-SNE method for AD/SQ classification.

In this section, we visualize the results obtained with both t-SNE and PCA dimensionality reduction methods (Figure 5 and Figure 6). Our analysis showed that the NS/SC dataset is much harder to classify than the AD/SQ dataset. In other words, the NS/SC data are more nonlinear than those in the AD/SQ dataset, and therefore, we applied the linear transformation method, PCA, on the AD/SQ dataset for dimensionality reduction, and the nonlinear method, t-SNE, on NS/SC for dimensionality reduction.

### 3.3. Metrics

In the Methods section, we included two validation methods: 5-fold cross-validation and independent dataset validation. A 5-fold cross-validation divides the whole dataset randomly into five folds. It uses four of them (80%) for training and one of them (20%) for testing. In contrast, an independent dataset validation method uses all the original data for training and an independent dataset, which comes from different papers, for testing.

In Table 2 and Table 3, we included test accuracy for the first-step NS/SC classification, the second-step AD/SQ classification, and the overall accuracy. The overall accuracy is calculated using the following formula:

(12) $$Overall_{acc}=SC\%\times NSSC_{acc}+NS\%\times NSSC_{acc}\times ADSQ_{acc},$$

where $Overall_{acc}$ is the overall accuracy; NS% and SC% are the percentage ratios of the numbers of non-small- and small-cell carcinoma cases to the total number of in our data *NSSC*%, respectively. 

Our overall accuracy comprises two parts: the NS/SC part and AD/SQ part. Because of the tree structure shown in Figure 2, the AD/SQ classification is a child of NS/SC classification, and therefore, their accuracy will be a product of $NSSC_{acc}\times ADSQ_{acc}$.

The percentages of *NS* and *SC* are used to obtain the weighted overall accuracy. In our dataset, 48.7% of NS and 51.3% are SC. $ADSQ_{acc}$ is the accuracy for AD/SQ classification and $\sum_{}^{}NSSC_{acc}$ is the accuracy for NS/SC classification. 

The last column showed the accuracy of using a naïve multiclass classifier, which directly classifies all four categories: SC, AD, SQ, and SC. 

In Table 2, it is demonstrated that both t-SNE and PCA dimensionality reduction methods give a higher accuracy than the multiclass classifier (0.920 ± 0.096 and 0.852 ± 0.063 compared with 0.76) and t-SNE gives the highest overall accuracy. In Table 3, we decided to add a different dataset, which includes fatty acid metabolites along with our original data from Table 2, to see whether our model could be generalized to other types of metabolites.

In addition to the cross-validation results shown in Table 2, Table 3 represents further results after running independent test sets on our model. This was performed to test the generalizability of our model. In Table 3, one can observe that accuracy does not drop significantly after including a dataset with fatty acid metabolites. This shows that our model is independent of the metabolite polarity. The overall accuracy only drops from 0.920 ± 0.096 to 0.902 ± 0.071 using the t-SNE dimensionality reduction and drops from 0.852 ± 0.063 to 0.812 ± 0.061 for PCA dimensionality reduction. Table 3 also concludes that t-SNE is a better dimensionality reduction method for metabolite-based lung cancer classification. 

## 4. Discussion

Compared with the naïve multiclass classifier (the test accuracy was below 70%), our method achieves significant improvement. By dividing them into several binary classifiers, each classifier achieves more than 90% accuracy and gives 92% overall accuracy. This significantly outperforms multiclass classifiers.

An observation in the Section 3 shows that the accuracy does not drop significantly after including additional fatty acids’ metabolites. This shows that our model can be further explored by datasets formed by various metabolites. Due to the limitations of our experiments, we could not test all metabolites related to lung cancers, but we believe that such a comparison between Table 2 and Table 3 is a good start. We will try to explore more metabolites in the future to see whether there are any possible improvements or constraints of our model. In the original dataset, metabolites focused on amino-acid biomarkers such as tryptophan, methionine, and proline, while other independent test sets included more fatty acid-derived molecules. 

We hypothesize that the difference in molecular weight and density generated molecular descriptors with different values that were not representative of our training data. Although there is such a huge difference in molecular descriptors, our model performs reasonably well on both. It will be interesting to observe how this model behaves on other metabolites such as nucleic acids.

### Why Is a Tree Structure Needed?

In this study, we proposed a novel architecture for the classification of lung cancer types combining the idea of hierarchical classification with that of a neural network. Neural networks and hierarchical classification have been widely used in cancer classification. We were inspired by Cerri’s and colleagues’ study of local hierarchical multiclass classification (HMC) and used this idea in cancer classification [37]. According to our knowledge, this is the first attempt to apply hierarchal multiclass classification on cancer metabolomics data. Since this was our first attempt, we chose a relatively simple two-stage task classifying adenocarcinoma (AD), squamous cell carcinoma (SQ), and small-cell carcinoma lung (SC) cancers. The first stage classifies cancers as either NS or SC and the second stage further classifies the NS class into the AD and SQ subclasses (Table 4).

Compared to traditional methods, which exclude hierarchical classification, our method splits a challenging multiclass task into smaller tasks and gives several advantages. First, the two-stage classifier is more accurate in the scenario of lung cancer classification. This benefit comes from the fact that lung cancers are not disjoint categories but biologically correlated with each other. Such a “divide and conquer” strategy allows our method to give much more accurate classification compared to traditional single multiclass classifier shown in Table 4.

The second advantage for two-stage classifiers is that they are “sensitive to local properties”. In other words, it is much easier to interpret which features are more important. A single multiclass classifier usually generates a complex matrix or tensor of weights that make it easy for human beings to understand the significance of each feature. In the case of a two-stage classifier, because each task is a very simple binary classifier, it is much easier to know which descriptors contribute the most. This advantage can help us relate to the machine-learning black box with biological metabolisms.

However, the main disadvantage of a two-stage classifier is that it is more computationally expensive than a single multiclass classifier. We have to train and test a cascade of neuron networks rather than a single neural network. Another main disadvantage is called error propagation, which means that errors made in the parent classifier will also contribute to its children. In our case, if we make a mistake in NS/SC classification, this will also affect our AD/SQ classification. However, in terms of overall accuracy, our two-stage classifier still has a much higher value than a single multiclass classifier. This means that the tradeoff is worthwhile in the case of lung cancer classification.

## 5. Future Work

In the cancer classification problem, we divided the whole task into several homogeneous classification subtasks and proposed a tree structure of neural networks to solve this problem. Such a strategy can be used to solve more complex data that may include multiple stages. We are also interested in other cancer classification problems without prior histopathology.

## 6. Conclusions

In this paper, we can diagnose the lung cancer type based upon patients’ metabolite profile. In our pipeline, we characterized metabolites by their molecular descriptors and then performed feature extraction and dimensionality reduction. We divided multiclass lung cancers into several binary classifiers, where each classifier is a small neural network. Our model achieves an overall test accuracy of 92.0%. Each classifier has more than 90% accuracy. Our two-stage classifier significantly outperforms the traditional naïve multiclass classifier by more than 14%.

## Figures and Tables

**Table 1:** Hyperparameters for neural network.

Hyperparameter	Value
Number of neurons NS/SC	[300, 400, 300]
Number of neurons AD/SQ	[300, 400, 300]
Dropout rate	0.5
Optimizer	Adam
Decay	Exponential decay
Decay rate and decay steps	0.999, 10,000
Learning rate	0.01
Epochs	500

**Table 2:** Cross-validation results for t-SNE and PCA dimensionality reduction methods.

Dimensionality Reduction Algorithm	First Step: NS/SC Classification Accuracy	Second Step: AD/SQ Classification Accuracy	Overall Accuracy	Naïve Multiclass Classifier
t-SNE	0.962 ± 0.04	0.911 ± 0.04	0.920 ± 0.096	0.76
PCA	0.923 ± 0.01	0.846 ± 0.09	0.852 ± 0.063	0.76

**Table 3:** Independent data test results for t-SNE and PCA dimensionality reduction methods.

Dimensionality Reduction Algorithm	First Step: NS/SC Classification Accuracy	Second Step: AD/SQ Classification Accuracy	Overall Accuracy	Naïve Multiclass Classifier
t-SNE	0.952 ± 0.03	0.895 ± 0.03	0.902 ± 0.071	NA
PCA	0.882 ± 0.03	0.842 ± 0.02	0.812 ± 0.061	NA

**Table 4:** Single multiclass classifier vs. two-stage classifiers.

	Single Multiclass Classifier	Two-Stage Classifiers
Best Overall Accuracy	0.760	0.920
Advantage	⁕ Less computation ⁕ Easy to implement	⁕ More accurate ⁕ Sensitive to local properties
Disadvantage	⁕ Less accurate (in cases of lung cancer dataset)	⁕ More accurate (in cases of lung cancer dataset) ⁕ Computationally expensive ⁕ Error propagation

## Data Availability

All data generated for this study are included in the article; further inquiries can be directed to the corresponding author. The data are not publicly available due to their availability from the other studies [8,11,14,15,16,17]. The code used in this study is available from the corresponding author upon reasonable request.

## Supplementary Materials

The following supporting information can be downloaded at: https://www.mdpi.com/article/10.3390/metabo13101055/s1, Table S1: Metabolites used as lung cancer biomarkers.

## Acronyms and Abbreviations

AD, adenocarcinoma; DNN, deep neural network; ES, enrichment score; FC, fold change; InfoGain, information gain; LC, large-cell lung cancer; LSTM, long short-term memory ML, machine learning; NS, non-small-cell lung cancer; PCA, principal component analysis; SC, small-cell lung cancer; SGD, stochastic gradient descent; SQ or SqC (used in other papers), squamous cell carcinoma; t-SNE, t-distributed stochastic neighbor embedding; WEKA, Waikato Environment for Knowledge Analysis; SC, small-cell carcinoma.
