# Supplementary material for: Two-Stage Deep-Learning Classifier for Diagnostics of Lung Cancer Using Metabolites
Source: Metabolites. 2023 Oct 7;13(10):1055. doi: 10.3390/metabo13101055 (PMC10609149; doi:10.3390/metabo13101055)
Supplement: Supplementary file 1 [file metabolites-13-01055-s001.zip › metabolites-2600439-supplementary.pdf]

Two-Stage Deep-Learning Classifier for Diagnostics of Lung Cancer Using Metabolites

Ashvin Choudhary, Jianpeng Yu, Valentina Kouznetsova, Igor Tsigelny, and Santosh Kesari

Supplemental Table S1. Metabolites used as lung cancer biomarkers (References numbers are from the main article).

Name of Metabolite	Type	Reference Number
1-Methylxanthine	SQ	8
1,6-Anhydroglucose	SQ	8
10-nonadecenoate	AD	8
12-HETE	SQ	8
17-methylstearate	SQ	8
2-deoxyadenosine 5-monophosphate	SQ	8
2-deoxyguanosine	AD	8
2-Hydroxybutyrate	SC	14
2-hydroxybutyrate	AD	8
2-methylsuccinic acid	AD	8
2-O-methylguanosine	SQ	8
2-piperidinone	SQ	8
3-hydroxybutyrate	SC	14
3-Hydroxydecanoate	AD	8
3-hydroxydecanoate	SC	14
3-Methyl-2-oxobutyrate	NS	8
3-methylhistamine	NS	8
3-phenylpropanoic acid	AD	8
4-acetamido-3-methoxyphenyl beta-D-glucopyranosiduronic acid	SQ	8
4-acetamidophenol	SC	14
4-hydroxyproline	SC	14
5-dodecenoate	NS	8
5-methylthioadenosine	AD	8
7-dehydrocholesterol	AD	8
8-Tocopherol	AD	8
a-glucopyranosiduronic acid	SC	14
a-hydroxybutyric acid	AD	8
acetoacetate	AD	8

acetylcarnitine	AD	8
acisoga	SC	14
adenosine 5-monophosphate	SC	14
adenosine 5'-monophosphate	NS	8
ADpSGEGDFXAEGGGVR	AD	8
adrenate	SC	14
Alanine	AD	8
alanylleucine	SC	14
alanylphenylalanine	NS	8
alanylphenylalanine	AD	8
arachidonate	AD	8
arachidonic acid	SQ	8
arginine	SQ	8
ascorbate	NS	8
aspartic acid	SQ	8
aspartyltryptophan	SC	14
atenolol	SC	14
azelate	SQ	8
bilirubin	AD	8
carnitine	AD	8
cellobiose	AD	8
cholate	SC	14
cis-inositol	NS	8
citric acid	AD	8
cortisol	NS	8
cysteine	SQ	8
dehydromucic acid	NS	8
delta-tocopherol	AD	8
DGLA	SQ	8
dipropylacetic acid	SQ	8
docosadienoate	SQ	8
docosadienoate	AD	8
docosapentaenoate	AD	8
docosatrienoate	SC	14
DSGEGDFXAEGGGVR	NS	8
eicosanoic acid	SQ	8
Eicosenoate	SC	14
eicosenoate	NS	8
eicosenoylglycerol	SC	14
erythritol	SC	14
erythronate	NS	8

galactonate	AD	8
galactose	SQ	8
gamma-glutamylcysteine	SC	14
gamma-tocopherol	SQ	8
glutamine-leucine	NS	8
glutathione	SQ	8
glycerol	SQ	8
glycerol 2-phosphate	SC	14
glycine	NS	8
glycochenodeoxycholate	NS	8
glycocholate	SQ	8
Hexanoylcarnitine	AD	8
hydroxybutyrylcarnitine	NS	8
hypoxanthine	AD	8
hypoxanthine	SQ	8
I-urobilinogen	AD	8
indoleacetylglutamine	AD	8
isoleucine	AD	8
isoleucylglutamine	NS	8
isoleucylserine	SC	14
laurylcarnitine	NS	8
laurylcarnitine	AD	8
leucine	SQ	8
leucylglutamine	SC	14
leucylglycine	SQ	8
linolenate	AD	8
lysine	SC	14
malonic acid	SQ	8
mannose	NS	8
methionine	SC	14
methionine	SQ	8
metoprolol	SQ	8
myo-inositol	SQ	8
N-(2-Furoyl)glycine	SQ	8
N-(2-Furoyl)glycine	AD	8
N-acetylaspartate	NS	8
N-Acetylmethionine	SC	14
N-Acetylneuraminate	NS	8
N-acetylputrescine	NS	8
N-ethylglycinexylidide	SC	14
N(1)-acetylspermine	SQ	8

N1-methyladenosine	AD	8
naproxen	AD	8
nonadecanoate	SC	14
octadecanedioate	SC	14
palmitoleic acid	NS	8
palmitoylcarnitine	SC	14
pelargonate	SQ	8
pelargonate	SQ	8
phenol	NS	8
phenylalanine	SC	14
phenylalanine	SQ	8
phenylalanylleucine	SQ	8
phenylalanylvaline	SC	14
phosphate	AD	8
pyroglutamine	SQ	8
quinate	AD	8
R-hydroxybutyric acid	SQ	8
ribitol	SQ	8
ribofuranose	NS	8
ribose	NS	8
ribose	SQ	8
salicylic acid	AD	8
serine	NS	8
serylleucine	SQ	8
sorbitol	AD	8
spermidine	NS	8
stearoyl sphingomyelin	SC	14
sulfate	SC	14
taurodeoxycholate	SQ	8
theobromine	SQ	8
theobromine	NS	8
theophylline	NS	8
threitol	SC	14
threonine	NS	8
threonylvaline	NS	8
thymine	SC	14
tocopherol	SQ	8
trimethylamine N-oxide	AD	8
trizma acetate	AD	8
tryptophan	NS	8
tryptophan	AD	8

tyrosine	NS	8
tyrosylleucine	NS	8
uridine 5-monophosphate	SQ	8
uridine diphosphate-N-acetylgalactosamine	SC	14
uridine monophosphate	NS	8
valine	AD	8
valylglycine	SQ	8
valylisoleucine	SQ	8
valylphenylalanine	NS	8
valylvaline	NS	8
xanthosine	AD	8
xanthosine 5-monophosphate	AD	8
